# How do social-economic differences in urban areas affect tuberculosis mortality in a city in the tri-border region of Brazil, Paraguay and Argentina

**DOI:** 10.1186/s12889-018-5623-2

**Published:** 2018-06-26

**Authors:** Marcos Augusto Moraes Arcoverde, Thais Zamboni Berra, Luana Seles Alves, Danielle Talita dos Santos, Aylana de Sousa Belchior, Antônio Carlos Vieira Ramos, Luiz Henrique Arroyo, Ivaneliza Simionato de Assis, Josilene Dália Alves, Ana Angélica Rêgo de Queiroz, Mellina Yamamura, Pedro Fredemir Palha, Francisco Chiaravalloti Neto, Reinaldo Antonio Silva-Sobrinho, Oscar Kenji Nihei, Ricardo Alexandre Arcêncio

**Affiliations:** 1 Nursing College of Ribeirão Preto, University of São Paulo, São Paulo, Brazil; 2 State University of West Paraná, Avenida Paraná, 1610, Foz do Iguaçu, Paraná, 85863-720 Brazil; 3 Department of Epidemiology, School of Public Health, University of São Paulo, São Paulo, Brazil

**Keywords:** Border crossing, Tuberculosis, Social inequity, Income, Continental population group, Races

## Abstract

**Background:**

The World Health Organization (WHO) launched the “End TB Strategy”, which aims to reduce tuberculosis (TB) mortality by 95% by 2035, Brazil has made a commitment to this, however, one challenge is achieving the goal in the border region, where the TB situation is more critical. The proposal was to analyse the spatial mortality due to TB and its socio-economic determinants in the general population, around the border areas of Brazil, Paraguay and Argentina, as well as the temporal trend in this region.

**Method:**

This ecological study considered the cases of TB deaths of residents of Foz do Iguaçu (BR), with its units of analysis being the census sectors. The standardized mortality rate was calculated for each area. Socioeconomic variables data were obtained from the 2010 Demographic Census of the Brazilian Institute of Geography and Statistics (IBGE). The scan statistic was applied to calculate the spatial relative risk (RR), considering a 95% confidence interval (CI). Spatial dependence was analysed using the Global Bivariate Moran I and Local Bivariate Moran I (LISA) to test the relationship between the socioeconomic conditions of the urban areas and mortality from TB. Analysis of the temporal trend was also performed using the Prais-Winsten test.

**Results:**

A total of 74 cases of TB death were identified, of which 53 (71.6%) were male and 51 (68.9%) people of white skin colour. The mortality rate ranged from 0.28 to 22.75 cases per 100,000 inhabitants. A spatial relative risk area was identified, RR = 5.07 (95% CI 1.79–14.30). Mortality was associated with: proportion of people of brown skin colour (I: 0.0440, *p* = 0.033), income (low income I: − 0.0611, *p* = 0.002; high income I: − 0.0449, *p* = 0.026) and density of residents (3 and 4 residents, I: 0.0537, *p* = 0.007; 10 or more residents, I: − 0.0390, *p* = 0.035). There was an increase in the mortality rate in people of brown skin colour (6.1%; 95% CI = 0.029, 0.093).

**Conclusion:**

Death due to TB was associated with income, race resident density and social conditions. Although the TB mortality rate is stationary in the general population, it is increasing among people of brown skin colour.

## Background

Tuberculosis (TB) is a serious public health problem worldwide, with one-third of the global population infected with Mycobacterium tuberculosis, which represents a large human reservoir [1]; the disease causes many deaths and leads to more poverty, notably in developing countries.

In South America, six countries account for 53.2% of all TB cases in the Americas, with Brazil having the largest percentage (33%), followed by Peru (13%), Colombia (5.6%), Bolivia (4.6%), Argentine (3.5%) and Venezuela (3.5%) [2, 3]. Although the incidence of TB in the general population is decreasing, there are still problems that hinder control, such as coinfection by TB and human immunodeficiency virus (HIV), a low rate of completion of treatment (close to 70%, while the WHO recommends 85%) and social inequality [1, 2].

Epidemiologically, the burden of TB is higher among the ethnic minorities, immigrants, people living with HIV, in extreme poverty, diabetics, people using drugs and those with mental disorders [2, 4–6].

Brazil is a signatory to the “End TB Strategy”, which foresees the elimination of TB by 2050 and a 95% reduction in mortality by 2035. Thus, one of the key issues is the trend of mortality among TB patients, particularly in the border regions where the disease is poorly controlled [7, 8]. Studies show that in border regions there are more episodes of abandonment, multidrug resistance and deaths due to TB, in addition to other neglected conditions, compared to non-border regions [9–13].

With regard to the health systems, Brazil adopts the universalist model that guarantees free diagnosis and treatment for TB patients and is the only country in Latin America to use this model. Argentina and Paraguay, although they have some differences between them, have fragmented health systems, with great importance given to the social insurance model of care for workers and beneficiaries, as well as public sector institutions that attend the uninsured and the private sector [14, 15]. These models of health-care delivery have implications regarding mortality among TB patients particularly in the border regions – that may delay the achievement of the targets under the “End TB Strategy” [1].

Literature about TB mortality in border regions and its determinants in Brazil is still limited. There are documented differences in socio-economic conditions resulting from cultural, political, language and ethnic variations which can negatively impact disease control and increase the risk of TB mortality. These epidemiological characteristics need to be further explored to better position the country to meet the “End TB Strategy” target for this indicator.

In this study, we aimed at analysing spatial mortality due to TB and its socio-economic determinants in the general population, around the border areas of Brazil, Paraguay and Argentina, as well as the temporal trend in this region.

## Methods

### Study type

This was an ecological study [16].

### Study scenario

**Fig. 1:**
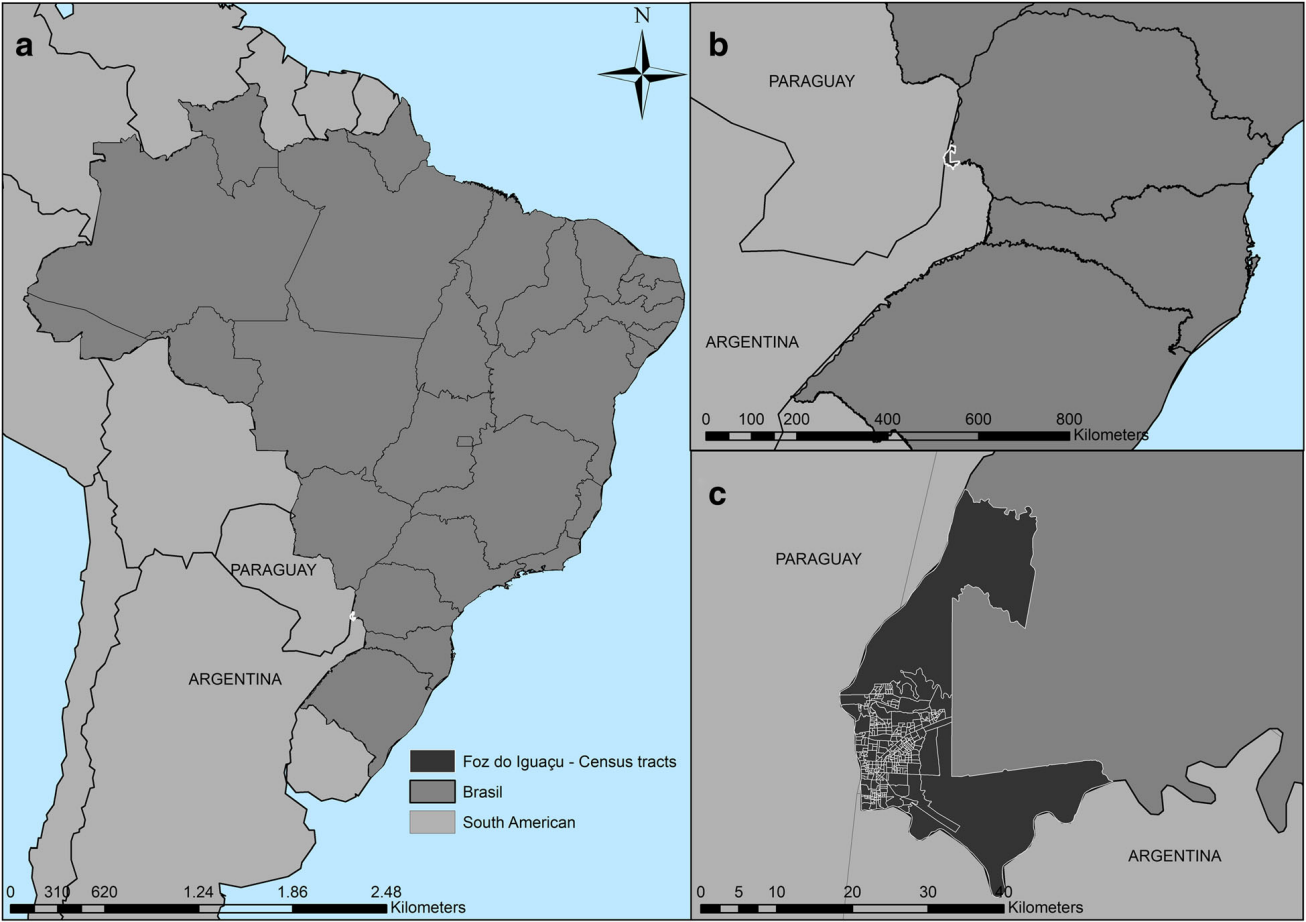
Map of the scenario. Legend: (**a**) Brazil; (**b**) Border region with Paraguay and Argentina; (**c**) Detail Municipality of Foz do Iguaçu

Argentina, Brazil and Paraguay, which constitute the countries of the tri-border area, present the following indicators, respectively: incidence rates of 25, 41, 41 per 100,000 inhabitants and mortality rates of 1.6, 2.7, 4.0 per 100,000 inhabitants [1]. The municipality of Foz do Iguaçu, as shown on the map (Fig. 1), is located on the tri-border, adjoining Ciudad del Este (Paraguay) and, to the south, Puerto Iguazu (Argentina) and has a population of 263,915 inhabitants of different ethnicities [17].

The presence of immigrants and tourists in the city creates a good ground for the transmission of communicable diseases in the population hence the higher demand for good-quality health services in the region [18].

The municipality is composed of 327 census sectors, 320 being urban and seven rural [19], with a Human Development Index (HDI) of 0.75; Gini index of 0.55; Poverty Incidence (proportion of people with low income) of 25.5%; a life expectancy at birth of 76.5 years; and a population coverage of the Primary Care Teams of 62.74% [20, 21].

It has 28 Primary Health Care (PHC) units, eight use the traditional model and 20 the Family Health Strategy. These units are administratively divided into five health districts and the municipality also has two emergency care units, two pre-hospital care services and one hospital that was maintained by the municipal administration until the end of 2015, and then started to be managed by the Paraná State Health Department in 2016 [22]. The study considered the census sectors of the urban area of the city of Foz do Iguaçu (BR).

### Study population

The study population was composed of deaths whose underlying cause was TB (A15.0 to A19.9, International Classification of Diseases – ICD) for people living in the municipality of Foz do Iguaçu, Paraná, Brazil, from 2004 to 2015.

### Data sources and study variables

The study used two sources of information: The Mortality Information System (SIM) and data from the 2010 Demographic Census of the Brazilian Institute of Geography and Statistics (IBGE).

The SIM variables selected were: date of diagnosis; date of birth; sex; municipality of the death; municipality of residence; street; number; neighbourhood; and cause of death. The variables obtained from IBGE were related to the “number of residents in the household”, “race/skin colour of the residents” and “per capita income of the residents” [19].

### Data collection

Data were obtained from the SIM of the Health Surveillance Department of the Municipal Health Department of Foz do Iguaçu, BR. The map of the census sectors was obtained from IBGE [19].

### Data analysis

Absolute and relative frequency measures were calculated for the categorical variables. For the age variable, position (mean and median) and dispersion (standard deviation) measures were calculated using the R Program version 3.3.2.

For each case, from the address of the residents in Foz do Iguaçu (PR), reference values for latitude and longitude were found, using the Google Earth™ Version 7.15 software. Then, the TerraView version 4.2.2 software was used to transform the latitude and longitude information into a shapefile format file of points, with SIRGAS2000 projection.

The union of the shapefile of cases with the shapefile of sectors was performed using the QGIS version 2.18 software, making it possible to identify, in addition to the distribution of the cases in the municipality, the census sectors in which they were included. Based on the information from the shapefiles and the 2010 Census, three spreadsheets (centroid, population and cases) were constructed using the Excel software to search for clusters, with the relative risk calculated using the SaTScan version 9.3 software.

The sweep spatial analysis technique was used, developed by Kulldorff and Nagarwalla (1995) [23], in which the search for risk clusters is performed by positioning a virtual circle of variable radius around each centroid and calculating the occurrence rate of the event within each virtual circle. If the observed value of the region delimited by the circle is larger than expected, it is called a risk cluster; if the value is lower than expected, it is called a low-risk or protective cluster, with this procedure being repeated until all centroids are tested [24].

For the identification of risk clusters, since TB deaths are countable variables and rare in relation to the population, the Poisson discrete model was used. The standard configuration applied by the SaTScan software adopted the following criteria: no geographic overlap of the clusters, maximum cluster size equal to 50% of the exposed population, circular-shaped clusters and 999 replications. Considering the low frequency of the event in the scenario studied, clusters with 10 and 5% of the exposed population were tested. This variation of the standard allowed the technique to find small clusters [25]. For the analysis of the scan statistic, the population was controlled for age and sex.

Under the conditions described, the analyses were purely spatial, spatio-temporal and spatial variation in the temporal trends, with the spatial relative risk (RR) and 95% confidence interval (CI) being calculated.

The RR refers to the analysis of a risk outcome within a geographically limited region (such as a country, municipality or census sector) [26], defined as the risk *λ*_*Z*_ in the region compared to the risk in all other regions [27].

$$ {\displaystyle \begin{array}{l}{\lambda}_z=\frac{E\left({Y}_z\right)}{E_z},\\ {}{E}_z=N\frac{P_z}{P_{+}},\end{array}} $$

where *Y*_*Z*_ is the Poisson random variable of the Z-region count, with the expected number given by E(Y_z_); P_Z_ is the population of region Z; P_+_ is the total population at risk in an area; and N is the total number of observed cases. In the same way *λ*_*A*\*Z*_ is defined. Thus, the true relative risk is given as [27]:

$$ RR=\frac{\lambda_Z}{\lambda_{A\backslash Z}}. $$

If both Z and A\Z have the same *λ*_*Z*_ = *λ*_*AZ*_ = *λ*, the relative risk is equal to 1. Assuming that Z is selected independently of the observed values, then the estimated relative risk is given by:

$$ {RR}^S=\frac{Nz\left|{E}_z\right.}{\left(N-{N}_Z\right)\left|{E}_A-{E}_Z\right.} $$

where N is the total number of cases, N_Z_ is the number of cases in cluster Z; E_A_ is the number of expected cases in the region under the null hypothesis; E_Z_ is the number of cases in the Z area under the null hypothesis. For the interpretation of RR^S^, when equivalent to 1, there is strong evidence that there is no risk cluster on the map; if below 1; tending to zero means low risk or area of protection; and above 1, represents the actual risk area and the likelihood [27].

The standardized rate of TB mortality (SRTBM) by sex and age for each census sector was estimated using Microsoft Excel 2010 and the result attached to the shapefile format file by QGIS version 2.18. The rate was calculated according to the following formula:

$$ SRTBM=\frac{\left(\sum \left(\left( Sd\times Pop\  standard- sector\right)/ Pop\  subgroup\right)/\sum Pop\  standard\right)}{\mathrm{T}} $$

where, “Sd” = number of study deaths;

*“Pop standard-sector” =* the population residing in the census sector of reference for the year 2010;

*“Pop subgroup”* = the population residing in the census sector divided into sex and age groups according to the median reference for the year 2010.

*“Pop standard*” = the standard population of the municipality of reference for the year 2010;

*T* = period studied in years. For this study, 12 years were considered.

The spatial dependence of the socio-economic conditions (“residents density”, “ratio of race/skin colour of residents” and “per capita income of residents”) were tested using the Global Moran Index (Moran I). It is important to highlight that spatial dependence is expressed by the following formula [28]:

$$ I=\frac{\sum \limits_{i=1}^n\sum \limits_{j=1}^n{w}_{ij}\left({z}_i-\overline{z}\right)\ \Big({z}_j-\overline{z\Big)}}{\sum \limits_{i=1}^n{\left({z}_i-\overline{z}\right)}^2} $$

where n is the number of areas; z_i_ is the value of the attribute considered in the area i; z bar is the mean value of the attribute in the study region; and w_i_ corresponds to the elements of the normalized spatial proximity matrix.

The Global Bivariate Moran Index was used to test the relationship between the socioeconomic conditions and the mortality rate [29]:

$$ I=\frac{Z^T CZ}{1^TC1} $$

where I is a variable-by-variable Moran correlation matrix; Z is a case-by-variable matrix whose elements are z-scored; C is a case-by-case binary connectivity matrix, and 1 is a case-by1 column matrix with all elements being 1 s.

This technique is the mean of the Bivariate Local Index of Spatial Association (Bivariate LISA, L) statistics. This Bivariate LISA technique is expressed for each area i from standardized values x_i_ of the mortality attribute in which [29]:

$$ {L}_{x,y}=\kern0.5em \frac{\sum_i\left[\left({\sum}_j{w}_{ij}\left({x}_j-\overline{x}\right)\ {\sum}_j{w}_{ij}\left({y}_j-\overline{y}\right)\right)\right]\ }{\left(\sqrt{\sum_i{\left({x}_i-\overline{x}\right)}^2}\ \sqrt{\sum_i{\left({y}_i-\overline{y}\right)}^2\ }\right)} $$

From the socio-economic conditions that were statistically significant for TB mortality, the Bivariate Local Index of Spatial Association (Bivariate LISA) was used, and Moran Maps were constructed for the study of the local autocorrelation. The bivariate LISA map (Moran Map) allowed the identification of the association of statistically significant values and comparison of local means [30]. The exit response respects the Moran scatter plot, in which the division of the four quadrants corresponds to the local spatial association patterns among the regions (X) and their neighbours (Yij).

In the bivariate analysis, when the index is positive, the relation is direct and the values are predominantly in quadrants 1 and 3, according to the following interpretation: in quadrant 1 the values are high-high (H-H), indicating, in this study, a region with high (above the mean) mortality rates surrounded by regions with high socio-economic condition values; in quadrant 3 the values are low-low (L-L), indicating a low-mortality rate region, in relation to the mean, surrounded by regions with low values in relation to the socioeconomic conditions studied. When the global index is negative, the relationship is inverted and the values are concentrated in quadrants 2 and 4, with quadrant 2 (low-high, L-H) showing, in this study, regions with mortality values below the mean surrounded by areas with high values in relation to the mean of the social condition analysed, and quadrant 4 (high-low, H-L) indicating regions with mortality values above the mean close to regions with low socio-economic condition values [30, 31].

The temporal trend of the TB mortality rate in the general population and by race/skin colour was also evaluated. Considering Y as the values of the temporal series and X as the time scale, the line of fit between the points in the time series that aims to estimate the trend is defined by the equation: Y = b_0_ + b_1_X. To reduce the heterogeneity of residual variances from the temporal regression analysis, the logarithmic transformation of the Y values [32] was applied. The analysis was performed with the Stata 13 statistical program using the Prais-Winsten self-reported analysis method. The result of this analysis was the annual percentage change, called the annual rate of increase, and its respective 95% CI. In the interpretation, the trend is considered to be decreasing if both values of the CI are negative; if these values are positive, there is an increasing trend; and a stationary trend when the CI contains the zero value [32, 33].

The Type I error α = 0.05 was set as statistically significant.

## Results

A total of 74 cases of TB deaths were identified in residents of Foz do Iguaçu (BR). However, four (5.4%) cases were excluded due to lack of address information and four other cases because the latitude and longitude were incomplete, which resulted in 66 (89.2%) geocoded cases.

Regarding the sociodemographic characteristics of the individuals who died from TB (Table 1), the mean age of the deaths was 50.7 (range 19–89), with males contributing 53 (71.6%), while the white coloured were 51 (68.9%). Thirty-four (45.9%) had obtained elementary education and 36 (48.6%) were single. The table shows the majority of deaths occurred in hospitals (*n* = 47; 63.5%); however, the causes of deaths were not confirmed by necropsy (*n* = 65; 87.8%), they were just based on medical reports (n = 65; 87.8%).

**Table 1:** Characteristics of deaths by tuberculosis of residents of Foz do Iguaçu (BR), 2004 –2015

Variable	N = 74	%
Age (years)		
0 to 19	1	1.3
20 to 59	46	62.2
60 or more	25	33.8
No information	2	2.7
Sex		
Female	21	28.4
Male	53	71.6
Skin color		
White	51	68.9
Brown	20	27.0
Black	3	4.1
Education		
Elementary	34	45.9
High school	25	33.8
Incomplete and Complete Higher	9	12.2
No information	6	8.1
Marital status		
Single	36	48.6
Married/Stable union	16	2.16
Divorced	6	8.1
Widowed	12	16.2
No information	4	5.4
Place of death		
Residence	9	12.2
Hospital	47	63.5
Other health facility	16	21.6
Others	2	2.7
Medical care		
Yes	64	86.4
No	9	12.2
No information	1	1.4
Necropsy		
Yes	7	9.5
No	65	87.8
No information	2	2.7

Source: SIM, Foz do Iguaçu, BR, 2016

Table 2 shows the clinical forms of TB deaths according to the ICD10, noting that the majority of cases were pulmonary TB (*n* = 67; 90.5).

**Table 2:** Basic cause of deaths by tuberculosis of residents of Foz do Iguaçu, 2004–2015

*Basic cause of death*	*N =* 74	%
Pulmonary tuberculosis	67	90.5
Extra-pulmonary tuberculosis	7	9.5

Source: SIM, Foz do Iguaçu, BR, 2016

**Fig. 2:**
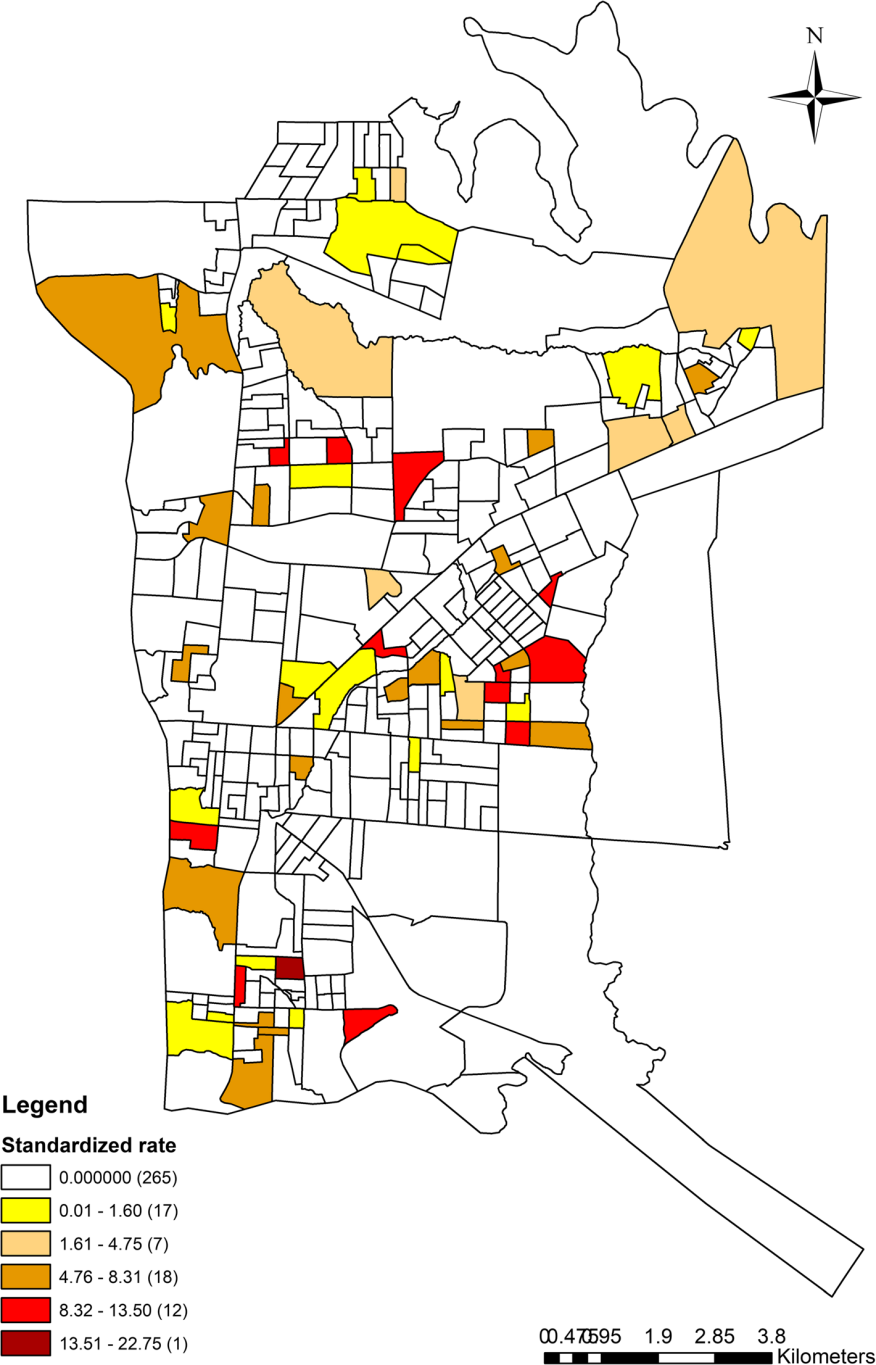
Standardized mortality rate for tuberculosis, Foz do Iguaçu (BR), 2004–2015

Fig. 2 presents the distribution of the standardized mortality rate for TB according to the census sectors, showing that this was not uniform throughout the municipality, with a variation that exceeded 10 times the mean rate of the municipality, 2.17 per 100 thousand (minimum difference from zero = 0.29, maximum = 22.75, mean = 0.95, standard deviation = 2.79).

**Fig. 3:**
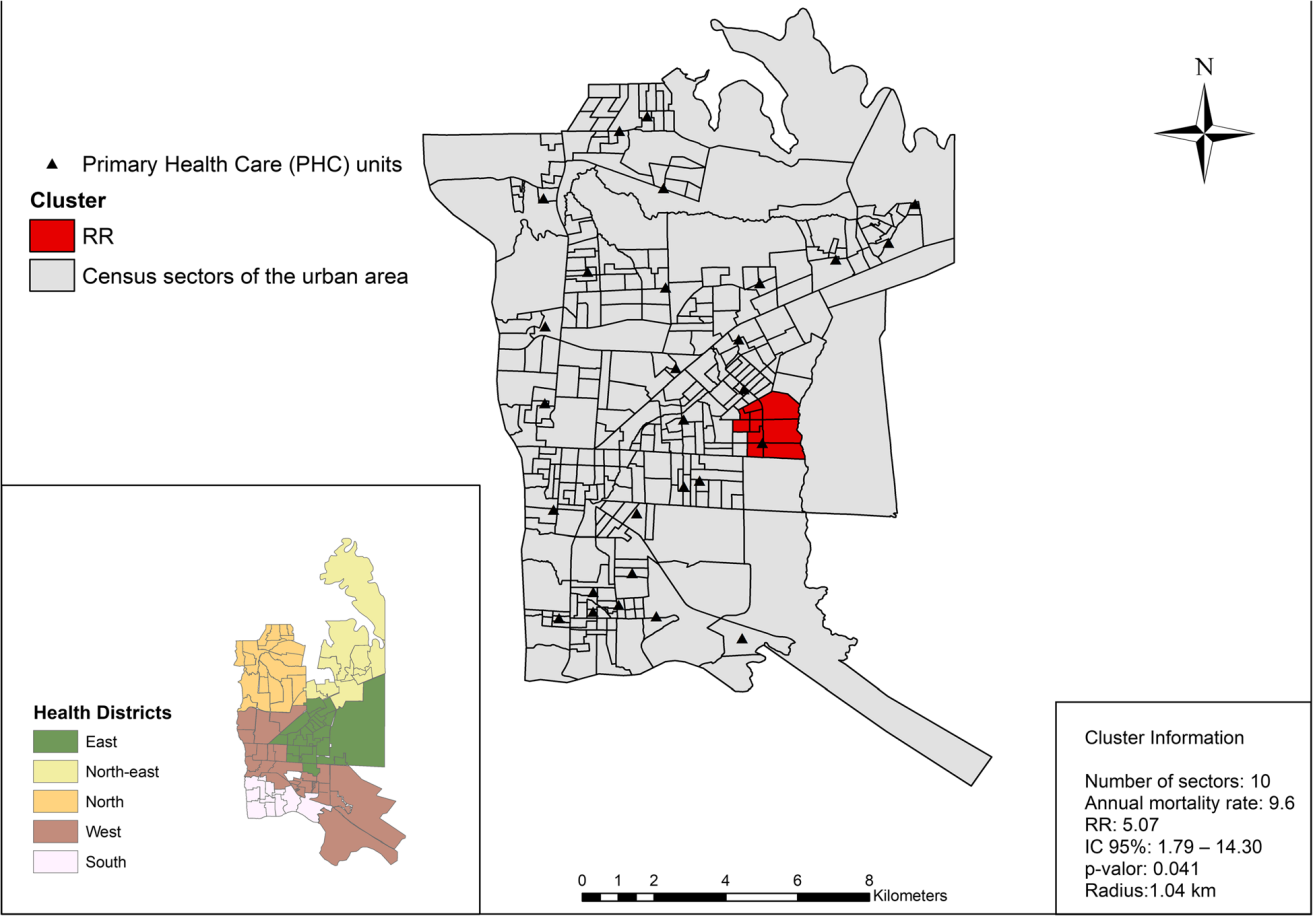
Health district and area with RR for tuberculosis mortality, Foz do Iguaçu (BR), 2004–2015

When applying the SatScan, a relative spatial risk area for TB mortality of RR = 5.07 (95%CI 1.79–14.30) was observed in the Eastern Health District (Fig. 3), which indicates that people in this region are 5 times more likely to die from TB than those of any other area of the municipality. Figure 3 shows the distribution of health units, with a family health unit registered in this area. The risk cluster was obtained by testing with 5% of the population.

Table 3 shows the mean, minimum and maximum of all independent variables tested by the Global Moran I and the Global Bivariate Moran I, which presented a statistically significant association with TB mortality.

**Table 3:** Spatial statistics the socioeconomic conditions and mortality from tuberculosis, Foz do Iguaçu (BR), 2004–2015

Variable	Mean	Min	Max	S	Global Moran I	*P*-value	Global Bivariate Moran I	*P*-value
*Proportion of residents according to Race / Skin colour*								
White	63.65	28.84	94.5	13.56	0.5396	0.001	−0.0432	0.031 *
Black	3.53	0	13.15	2.33	0.2316	0.001	0.0276	0.109
Asian	1.41	0	6.94	1.30	0.1930	0.001	−0.0376	0.043*
Brown	31.24	2.88	66.14	13.03	0.5056	0.001	0.0440	0.033*
Indigenous	0.16	0	3.67	0.36	0.0171	0.321	−0.0097	0.343
*Proportion of households by monthly income* per capita								
Up to 1/8 minimum wage^a^	4.87	0	27.22	4.52	0.2302	0.001	−0.0611	0.002 *
10 or more minimum wages***	1.09	0	17.64	2.34	0.4845	0.001	−0.0449	0.026 *
*Household density*								
Proportion of households with 3 or 4 residents	47.75	21.60	80.00	6.41	0.2820	0.001	0.0537	0.007 *
Proportion of households with 10 or more residents	0.29	0	3.26	0.52	0.1562	0.001	−0.0390	0.035 *

*s* standard deviation, *statistically significant variables; ^a^Approximately US$1.00 per day; ***Approximately US$96 per day

**Fig. 4:**
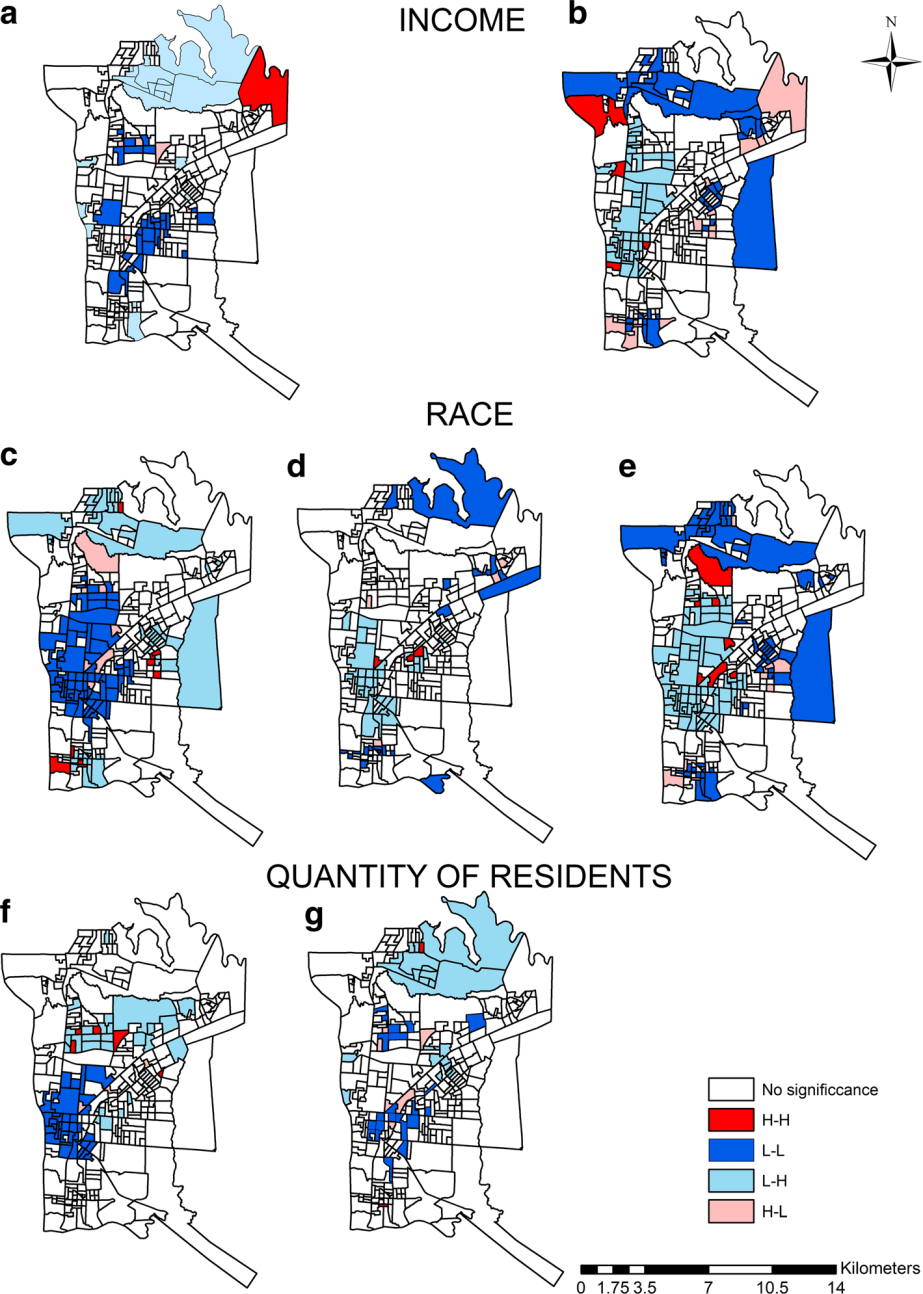
Bivariate LISA Map of socioeconomic conditions and mortality rate from tuberculosis, Foz do Iguaçu, 2004–2015. Legend: **a** - Association with Proportion of residents with per capita income of up to 1/8 minimum wage; **b** - Association with Proportion of residents with per capita income of 10 or more minimum wages; **c** - Association with Proportion of residents of brown skin color; **d** - Association with Proportion of residents of Asian race; **e** - Association with Proportion of residents of white skin colour; **f** - Association with Proportion of households with 3 or 4 residents; **g** - Association with Proportion of households with 10 or more residents

Next, the bivariate LISA was applied for the socioeconomic conditions with a statistically significant association with TB mortality (Fig. 4).

The results from the LISA application are shown in Fig. 4. According to Fig. 4a, it was possible to verify that the low-low (L-L) pattern was present in the East and Centre-West regions of the municipality, which means that areas with low mortality rates are surrounded by areas with a low proportion of residents with low per capita income (Fig. 4a). Figure 4a also shows that the high-high pattern (H-H) occurred in a peripheral sector of the North-east region, indicating an area with a high mortality rate close to sectors with a high proportion of households with low per capita income.

When the income variable of 10 minimum wages (MW) or more (4B) was analysed, the high-high (H-H) areas were scattered between the Mid-west and North regions, which means that areas with a high TB mortality rate were close to sectors with high proportions of households with the income mentioned. Furthermore, areas with an L-L pattern remained on the periphery and were present in almost all the regions.

Regarding the L-H pattern, Fig. 4a shows that this condition was more concentrated between the North and North-east regions, indicating a low mortality rate where there is a high percentage of income per capita of up to 1/8 minimum wage, whereas in Fig. 4b this pattern remains among the North and Central-West regions, showing sectors with a low TB mortality rate surrounded by areas with a high proportion of residents with a per capita income of 10 minimum wages or more.

In Fig. 4c, d and e, which express the brown/Asian/white race/skin colour, respectively, it is possible to observe that the H-H pattern for the first is in the North, South and East regions, with a large cluster of the L-L pattern in the centre of the map; and for the second, an L-L pattern with several sectors dispersed between the North, Northeast and South regions. The H-H pattern appears in three sectors in the East and Central-West regions. The L-H pattern can also be seen to be concentrated in the Central-West and East regions, indicating a low mortality rate and high proportion of Asian race residents. With regard to Fig. 4e, it can be seen that the L-L pattern is more present in the periphery of the municipality; whereas the H-H pattern is found in seven sectors, being dispersed and located in the East, Central-West and North regions. A large L-H cluster pattern is also located between the East, Central-West and North regions.

In Fig. 4f, referring to the association with the proportion of households with 3 or 4 inhabitants, a large cluster with an L-L pattern was found between the North, East and North-east regions; there were also, to a lesser extent, sectors with an H-H pattern dispersed in the North. Figure 4g, “Proportion of households with 10 or more inhabitants”, shows a sector with H-H pattern in the North region, with the L-L pattern occurring in a dispersed way. The L-H pattern presents a cluster between the North and Northeast regions, another cluster in the East region and two sectors dispersed in the Central-West region.

**Fig. 5:**
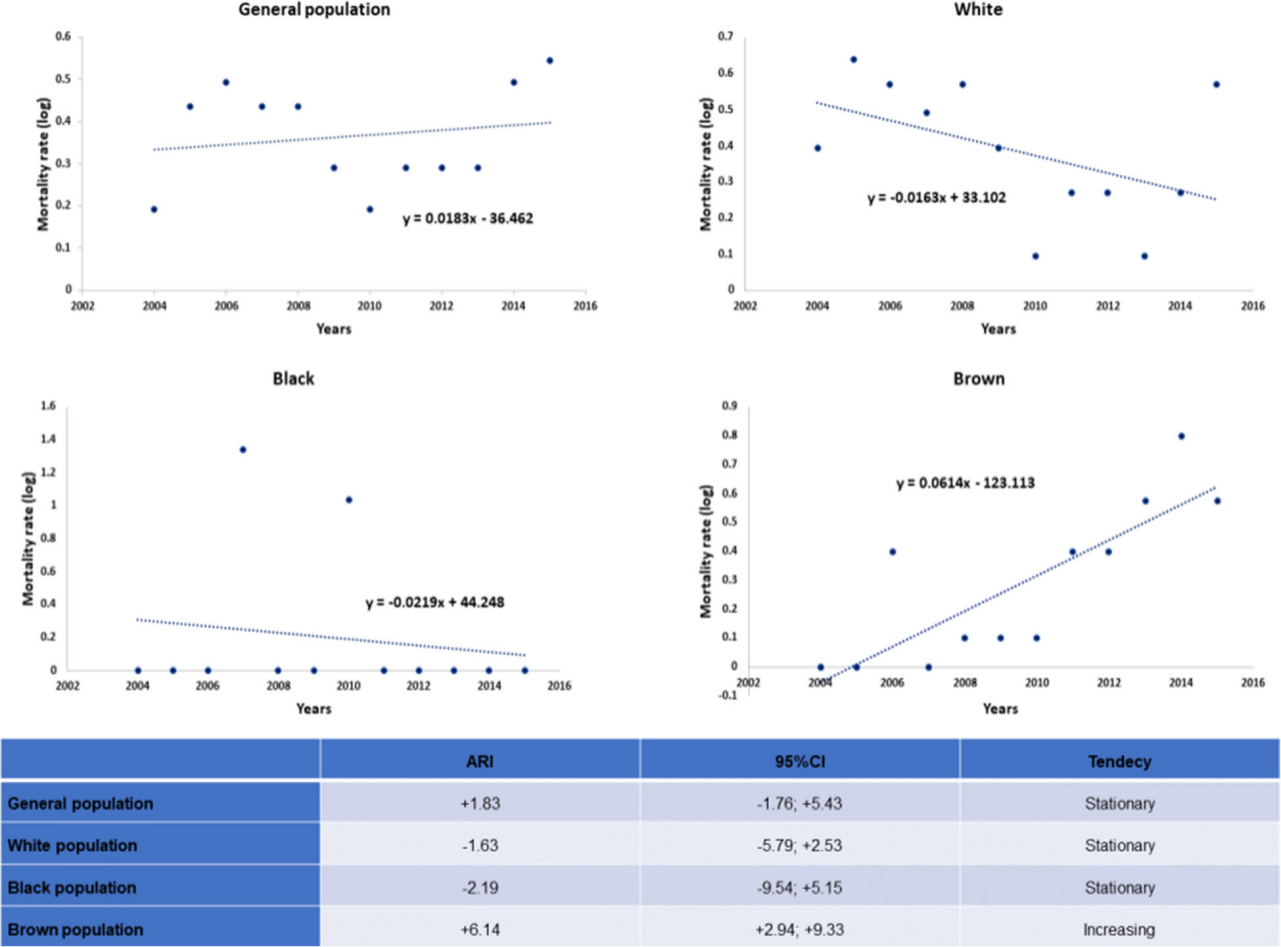
Temporal trend of the tuberculosis mortality rate, Foz do Iguaçu (BR), 2004–2015. Legend: ARI = annual rate of increase (percentage); 95% CI = 95%confidence interval (percentage); Trend = interpretation of the trend

Figure 5 shows the temporal trend of TB mortality in the general population and considering people of white, brown and black skin colour. From this figure it is possible to observe that brown skin colour stands out, as in these people TB mortality presents an annual growth of 6.14%, while for the others this is stationary.

## Discussion

The study sought to identify risk areas for TB mortality and how socioeconomic differences affect this event and its temporal trend in a municipality located in a tri-border region. Through the study, it was possible to identify a TB mortality risk area, with this being concentrated in a region with less favourable socio-economic conditions. It was observed that income, race, and density of household residents presented statistically significant spatial associations with TB mortality. Regarding the temporal trend, it was observed that death among TB patients in the general population had not changed significantly over the past 12 years, therefore, it remained stationary.

In relation to the profile of cases of death due to TB in a tri-border municipality, a predominance of males, with elementary education and single individuals was observed, which was also found in other studies performed in Brazil [7, 34, 35]. However, contrary to the other Brazilian studies, a difference in this study was the predominance of white people, which can be attributed to the historical process of migration in this region, which consisted of, in the majority, Europeans [21, 36]. This has resulted in the population of the municipality being mostly white. In a way, the general profile (%) of the deaths follows the general profile of the local population [21]. When conducting the bivariate analysis and the temporal trend, there was a difference in the behaviour of TB death in relation to the race/skin colour category.

A risk area for TB mortality of 5.07 (95%CI = 1.79–14.30) was observed when compared to the other areas. There is a health unit with a Family Health Strategy (model Primary Health Care) located in the area, however, this result highlights the access to this service for TB patients or the limitations of this unit in overcoming the social inequalities that surround it. Although the Pan American Health Organization (PAHO) has stimulated the renewal of Primary Health Care (PHC) in Latin America, including the border regions, to promote equity and human development [37], there is fragility in the scope of this proposal, since the PHC teams are often unable to cover the entire population.

It is worth noting that the risk of mortality in our study matches the difference in socio-economic conditions as previously reported [38–40]. We found that residing in an area of inequality carries a 5-fold risk of death as compared to areas with more favourable conditions.

In relation to the socioeconomic conditions associated with mortality (Table 3), represented by the LISA test, variations were observed in terms of socioeconomic conditions and how they affect TB mortality. An interesting result has been revealed in this study, and that the negative association between people who lived on up to 1/8 of minimum wage (MW) and TB mortality. The opposite result would have been expected as tuberculosis classically affects populations with lower income. Maybe these findings are related to social programmes such as the cash transfer named the “Bolsa Familia Programme” launched by the Brazilian government (2003) in recent decades and have removed people from extreme poverty and therefore offering social protection [41].

According to the Brazilian rules, the Bolsa Familia Programme is a conditional cash transfer programme through which parents receive a fixed monthly stipend (in this case R$70, about $ 30) in exchange for sending their children to school and complying with different health checkups [42]. This programme has achieved reduction of poverty by half from 9.7 to 4.3%, due to its broad scope and coverage, which represents 50 million low-income Brazilians or a quarter of the total population [43, 44].

Another study, when discussing social income transfer programmes, considered that, even though they are not specific for TB, their benefits contribute to combating the disease [43]. In Brazil, the Bolsa Familia Program is conditional on the attachment of the family to the primary health network, the education network, and employment and income generation programmes [45], which may impact on social determinants related to poverty or neglected diseases such as TB [44].

A favourable situation observed was the negative association between the per capita income of 10 MW or more and TB deaths, which evidences protection (Table 3). This result was expected in the study, since the income is a social determinant widely explored in studies previous [45]. There are different interpretative models of the social determinants, and specifically the neo-materialist approaches have emphasized economic status as a determinant of the production of health and disease, assuming that differences in income is exclusively what determines the access to good or weak services of education, transport, sanitation, housing, health services [46]. Although it may be worth understanding this specific determinant, nevertheless only analysing the TB from this perspective is a really poor and limited approach.

While there is a vast amount of literature about the relationship between TB and income, one very peculiar outcome was the identification of the H-H pattern for this association (Fig. 4b), which suggests regions of high social inequality and also high TB mortality. Therefore, the findings evidence that income is not the great determinant of TB deaths, because there were area with low income and high income equally affected by disease.

Researchers have advanced in the discussion of a model of social determinants based on the theory of social capital. They affirm that there is the development of the network of links and support, as well as associations between individuals and groups, even in unequal living conditions [46]. This a specific situation that is very common in areas affected by poverty, where people learn to help each other to survive, as happened, for example, in Europe in the post-war period. However, this process may not be found in areas with higher incomes, which makes them more vulnerable, perhaps this may explain the results.

The findings showed there was a positive relationship with the condition of brown race/skin colour, which means that as this proportion increased, in a given census sector, the mortality rate due to TB also increased in its neighbours. For the proportions of residents of Asian race and white skin colour, the relationship of the association was inverted. There is no plausible biological relationship in the scientific literature to support this difference, however, the construction of Brazilian society [47] and more specifically the history of the study region should, be considered.

There is historical information that shows that at some point in its formation, the city was colonized by people of European descent [36], which explains why more than half of the current population have the white skin characteristic and those with brown skin compose less than one-third of the population [21].

More recently, in the 70s, due to the construction of a hydroelectric plant on the Paraná River, which separates Brazil from Paraguay, the municipality of Foz do Iguaçu received a large number of immigrants [48]. They were construction workers and their families, coming from other Brazilian regions, which allowed the miscegenation of the region. This fact and the development of the city in the following decades should be considered in order to understand how the new neighbourhoods were organized and which residents lived there. The way in which people reorganized themselves in space appears as a hypothesis for the mortality rate of TB, since the groups did not mix equally within the whole territory. This would justify high rates in the regions with the highest percentage of residents of brown colour.

The analysis of the temporal trend shows that mortality was more prominent among the people of brown skin colour since, while the mortality rate decreased among other groups (white and black) and some did not have any cases recorded in the period (indigenous and Asian), this group showed an increase.

The issue of race/skin colour has also been presented in other studies as a trait of social inequality, with marked differences in Brazil in terms of opportunities for white and black or brown people, to the point of needing to establish racial quota policies in order to reduce differences. The population of black or brown skin colour is also generally more affected by violence and suffers most from poverty. These people also have little political representation, less access to education and higher education and have a lower average income than white people [49].

A study performed in Michigan (USA) also highlighted the disparity in the incidence of TB cases when comparing races and nationalities. In this study, black people had a mean incidence rate 25 times higher than whites and Asians had an incidence rate 19 times higher than whites [50]. These data demonstrate that TB illness and death are also socially modelled.

A curious fact is that the black race presented no relationship with mortality due to TB, which may be due to the miscegenation in the region, thus, there are more people of brown colour than black. However, black and brown people, according to a study in the Brazilian context, lived in worse conditions in 2006 than the majority of white people, with them representing 66% of the country’s poverty [51], which is related to the results of this study. In Brazil, obtaining racial and skin colour data of the population occurs in a self-declared way, since it is the subjects themselves who attribute to themselves a racial and colour identity according to the options provided by the IBGE enumerator. However, in relation to the act of recording the death, it is the attending physician who completes this information on the death certificate.

What this association shows, however, is that because of the way social relations are constructed in this community, illness and death do not occur equally among racial groups; that is, the development of social relations between these groups produced inequality between them, which, in turn, produces vulnerability for one or more groups, and in the context studied, leads to TB death. Corroborating, a study conducted in China, the results indicated that residing in a border region and being an ethnic/racial minority had an association with TB mortality [52].

In relation to the number of residents per household, the percentage of households with 3 or 4 residents presented a positive association with socioeconomic status, while the percentage of households with more than 10 residents presented a negative association, which is in disagreement with the studies that reported that the higher the household density, the higher the risk of illness [53, 54]. One hypothesis for this finding is that the mean number of residents per household was 3.2 (IBGE, 2010) [19] and that in Latin America, large families seem to exert a protective effect on individuals’ health, which was evidenced in a study with older adults and may also be true for TB [55]. Larger “family” arrangements or families neighbouring these would have better conditions for caring for patients, avoiding mortality, while in smaller family arrangements or where the proportion of these arrangements is greater, it becomes more difficult to provide care.

It is worth noting that social inequality is a phenomenon that mainly affects developing countries, especially marked by diseases of poverty such as TB, where there is no harmony in the standard of living of the population, with regard to the spheres of economics, education, profession, gender, race and/or colour, which impacts their health indicators.

In Brazil, greater social inequality is governed by economic inequality, where income is distributed heterogeneously in society, with most of this income being concentrated among some people, to the detriment of others living in extreme poverty, which has a significant impact on the TB mortality rate. However, the study showed that living in extreme poverty had no relation to TB mortality, which can be attributed to government programmes such as the Bolsa Família Programme, which has helped remove thousands of people from extreme poverty and thus avoided deaths.

There are other types of inequalities, such as the social condition discriminated by race/skin colour, which means that some groups have fewer opportunities than others, notably the people of brown skin colour. This was evidenced through its relationship with TB mortality in this border region. Generally, these opportunities relate to basic education and higher education, employment and lack of incentives for social mobility [49]. According to the results of this study, there is an increasing trend in the mortality rate, which may be related to racial inequality in Brazil.

Working with secondary data can be considered a limitation of this study, as there is information bias and incomplete or incorrect data, which is dependent on the quality of the registration by the person. Another gap refers to the death verification system itself, in which there may be underreporting of deaths due to TB. A further limitation is due to the fact that the study considered only urban areas, due to the difficulty of processing rural data and information.

In addition, the IBGE database [19] with the census sectors presented a restricted number and type of variables and did not allow an interface with other information systems. Because it is a border region, it is difficult to obtain access to health information from the neighbouring countries, especially of the same level of quality or from similar information systems, so that some kind of comparison would be possible.

However, the study advances knowledge by raising important aspects of TB mortality in a border region. With regard to the aim of reducing TB mortality by 95% by 2035, this proposal should be linked to reducing the inequalities observed in these regions, correcting the inequity in opportunities, notably for people of brown skin colour and those without access to health services.

## Conclusions

According to our findings it can be concluded that the risk of TB mortality in the tri-border region of Brazil is high with variability among various locations. The socioeconomic conditions associated with TB mortality include income status, resident density and race/skin colour. We may speculate that the inverse relationship between economic status and mortality is due to other confounding factors in the population that mainly target the poor communities such as the programmes of income redistribution conditional to health programmes. The population with brown skin colour were more likely to die compared to their black and white counterparts. Contrary to what has been established elsewhere, the high density of household members was inversely associated with TB mortality.

## Abbreviations

BR: Brazil
CI: Confidence interval
HDI: Human development index
HIV: Human immunodeficiency virus
IBGE: Brazilian Institute of Geography and Statistics
ICD: International classification of diseases
LISA: Local Bivariate Moran I
MW: Minimum wage
PAHO: Pan American Health Organization
PHC: Primary Health Care
RR: Relative risk
SIM: Mortality Information System
SRTBM: Standardized rate of TB mortality
TB: Tuberculosis
WHO: World Health Organization
